# Supplementary material for: In-Depth Analysis of an Obligate Anaerobe Paraclostridium bifermentans Isolated from Uterus of Bubalus bubalis
Source: Animals (Basel). 2022 Jul 9;12(14):1765. doi: 10.3390/ani12141765 (PMC9311886; doi:10.3390/ani12141765)
Supplement: Supplementary file 1 [file animals-12-01765-s001.zip › animals-1671273-supplementary.pdf]

Title In-depth analysis of an obligate anaerobe *Paraclostridium bifermentans* isolated from uterus of *Bubalus bubalis*

Purva Gohil1, Kajal Patel 1, Srushti Patel 2, Ramesh Pandit 1, Vishal Suthar 3*, Srinivas Duggirala2, Madhavi Joshi 1, D B Patil 3and C G Joshi 1

1 Gujarat Biotechnology Research Centre, Gandhinagar, Gujarat- 302010

2 Department of Microbiology, Gujarat Vidyapith

3 Directorate of Research, Kamdhenu University, Gandhinagar, Gujarat, India-382010

*Corresponding author:

Vishal Suthar. Email: vsuthar28@gmail.com

M: +919824491049

Directorate of Research, Kamdhenu University

Gandhinagar, Gujarat, India-382010

Table S1. Predicted genes from the whole genome with different gene-finding tools.

Program	No. of genes predicted
Prodigal	3495
Glimmer-3	3521
GenemarkS+2	3,511
MetaGeneAnnotator	3456

Table S2. Genome annotation results with different platforms.

Feature	PROKKA	RAST	DFAST	PGAP (NCBI)
Genes	3449	3521	3456	3511
rRNAs	5	6	1	3
tRNAs	55	54	36	55
CRISPERS	0	0	0	0

Table S3. Digital DDH values for pairwise comparisons of user genomes vs. type strain genomes.

Query strain	Subject strain	dDDH (in %)	G+C content difference (in %)
Paraclostridium bifermentans GBRC	*Paraclostridium dentum* SKVG24 T	82.7	0.53
Paraclostridium bifermentans GBRC	*Paraclostridium bifermentans* subsp. *muricolitidis* CCUG 72489	80.5	0.78
Paraclostridium bifermentans GBRC	*Paraclostridium bifermentans* ATCC 638	72.5	0.43

Paraclostridium bifermentans GBRC	*Paraclostridium benzoelyticum* JC272	66.6	0.57
Paraclostridium bifermentans GBRC	*Paeniclostridium ghonii* DSM 15049	39.3	1.06
Paraclostridium bifermentans GBRC	*Paeniclostridium sordellii* ATCC 9714	31.5	1.43
Paraclostridium bifermentans GBRC	*Romboutsia ilealis* CRIB	27.4	0.96
Paraclostridium bifermentans GBRC	*Romboutsia hominis* FRIFI	26.4	0.51
Paraclostridium bifermentans GBRC	*Clostridium dakarense* FF1	26.2	0.89
Paraclostridium bifermentans GBRC	*Romboutsia lituseburensis* DSM 797	26	1.18

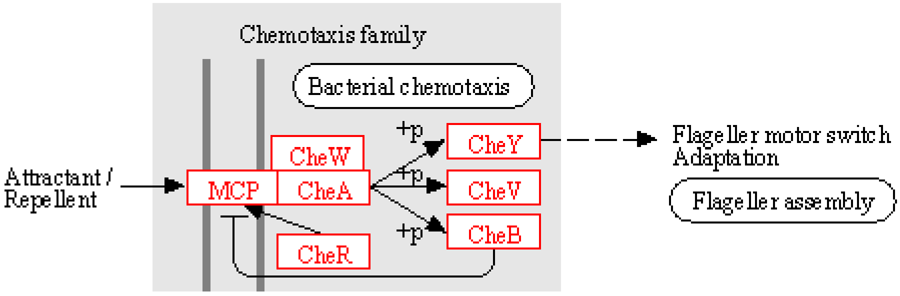

Figure S1. Flagellar assembly of strain GBRC in a hydrophobic environment where cheW is purine-binding chemotaxis protein, MCP; aerotaxis receptor, CheA; two-component system, CheR; chemotaxis protein methyl transferase, CheB; Protein-glutamate, CheV; two-component system.

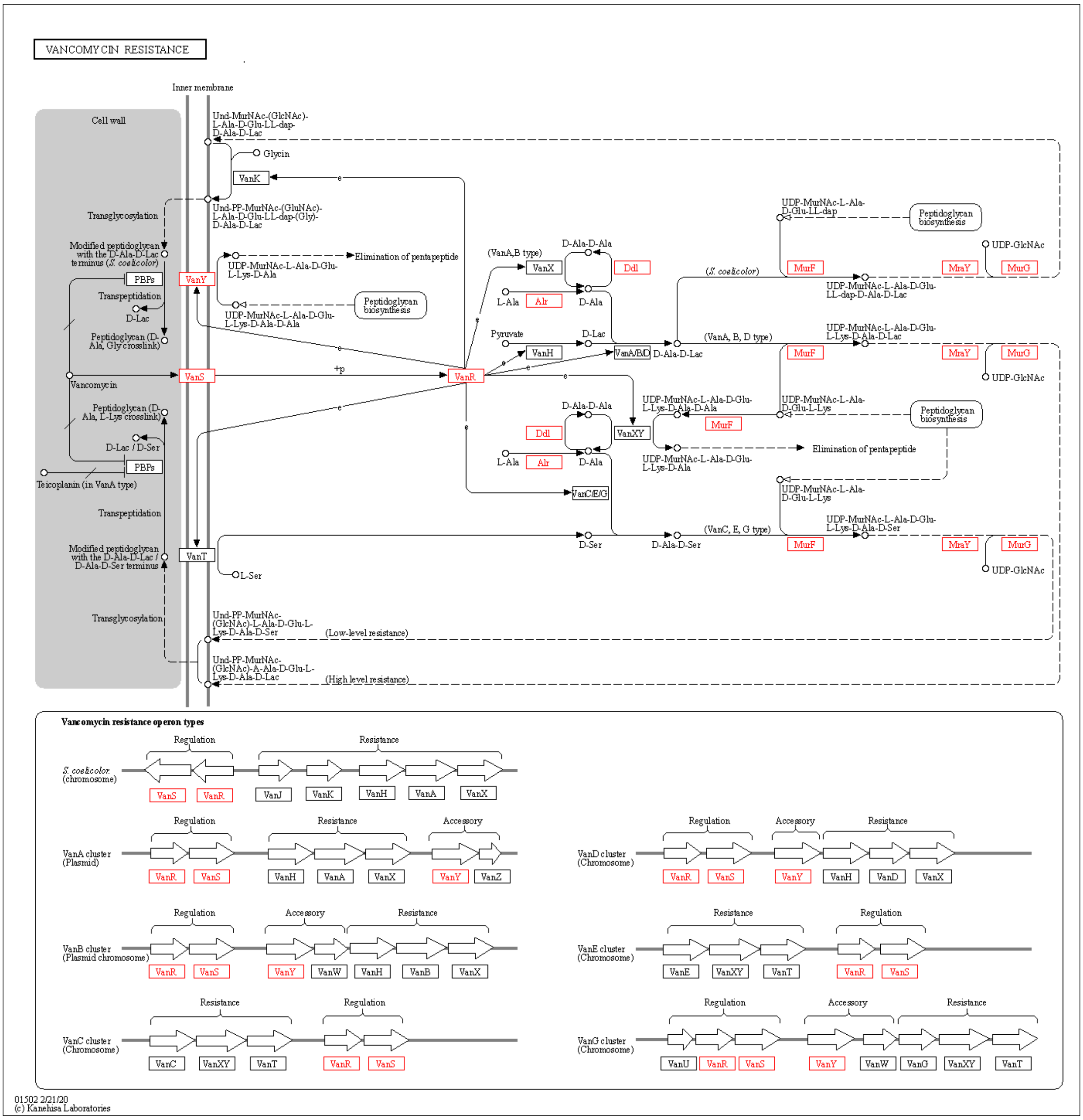
VANCOMYCIN RESISTANCE
Inner membrane
Cell wall
Und-MurNAc-(GlcNAc)-L-Ala-D-Glu-LL-dap-D-Ala-D-Lac
Glycin
VanK
Und-PP-MurNAc-(GluNAc)-L-Ala-D-Glu-LL-dap-(Gly)-D-Ala-D-Lac
Transglycosylation
Modified peptidoglycan with the D-Ala-D-Lac terminus (S. coelicolor)
PBPs
Transpeptidation
D-Lac
Peptidoglycan (D-Ala, Gly crosslink)
VanY
UDP-MurNAc-L-Ala-D-Glu-L-Lys-D-Ala
Elimination of pentapeptide
Peptidoglycan biosynthesis
UDP-MurNAc-L-Ala-D-Glu-L-Lys-D-Ala-D-Ala
Vancomycin
VanS
+p
VanR
Peptidoglycan (D-Ala, L-Lys crosslink)
D-Lac / D-Ser
Teicoplanin (in VanA type)
Modified peptidoglycan with the D-Ala-D-Lac / D-Ala-D-Ser terminus
VanT
L-Ser
Und-PP-MurNAc-(GlcNAc)-L-Ala-D-Glu-L-Lys-D-Ala-D-Ser
(Low-level resistance)
Und-PP-MurNAc-(GlcNAc)-A-Ala-D-Glu-L-Lys-D-Ala-D-Lac
(High level resistance)
(VanA,B type)
D-Ala-D-Ala
VanX
Ddl
L-Ala
Alr
D-Ala
Pyruvate
VanH
D-Lac
VanA/B/D
D-Ala-D-Lac
(S. coelicolor)
(VanA, B, D type)
UDP-MurNAc-L-Ala-D-Glu-LL-dap
MurF
MraY
MurG
UDP-GlcNAc
UDP-MurNAc-L-Ala-D-Glu-LL-dap-D-Ala-D-Lac
UDP-MurNAc-L-Ala-D-Glu-L-Lys-D-Ala-D-Lac
VanXY
UDP-MurNAc-L-Ala-D-Glu-L-Lys-D-Ala-D-Ala
UDP-MurNAc-L-Ala-D-Glu-L-Lys
UDP-MurNAc-L-Ala-D-Glu-L-Lys-D-Ala
VanC/E/G
D-Ser
D-Ala-D-Ser
(VanC, E, G type)
UDP-MurNAc-L-Ala-D-Glu-L-Lys-D-Ala-D-Ser
Vancomycin resistance operon types
Regulation
Resistance
Accessory
S. coelicolor (chromosome)
VanS VanR VanJ VanK VanH VanA VanX
VanA cluster (Plasmid)
VanR VanS VanH VanA VanX VanY VanZ
VanB cluster (Plasmid chromosome)
VanR VanS VanY VanW VanH VanB VanX
VanC cluster (Chromosome)
VanC VanXY VanT VanR VanS
VanD cluster (Chromosome)
VanR VanS VanY VanH VanD VanX
VanE cluster (Chromosome)
VanE VanXY VanT VanR VanS
VanG cluster (Chromosome)
VanU VanR VanS VanY VanW VanG VanXY VanT
01502 2/21/20
(c) Kanehisa Laboratories

Figure S2. KEGG pathway for vancomycin resistance mechanism
